# The Dublin School of Surgery

**Published:** 1863-10

**Authors:** 


### Art. V.—THE DUBLIN SCHOOL OF SURGERY.

The Dublin School of Medicine and Surgery is familiarly known throughout Europe. Many distinguished men have contributed to its reputation. To few has it been more indebted than to the eminent author of *The Reports in Operative Surgery,* to which attention is invited. These reports extend over a series of years. They record many of those remarkable cases and their treatment which have ranked Mr. Butcher amongst the most successful illustrators of the high art of his profession. They also exhibit him as a clinical teacher whose experience is ever prompt to eliminate from hospital observation great principles for the guidance of others in cases of difficulty or emergency. The operations they detail are of a character best described by the term "capital," in which the alternative between positive death and possible recovery is determined in favour of the latter by the judicious and often daring use of the knife. While a perusal of those all but hopeless cases in which a successful treatment has been achieved, is calculated to deeply impress the reader with the extraordinary dexterity of Mr. Butcher in everything that appertains to operative surgery, it is when their subsequent treatment is considered, and his observations on their diagnosis analysed, that his genius for the healing art is best proclaimed.

The first "Report" includes a series of cases in which "wounds of the principal arteries" are treated of. When it is advisable to tie both ends of the bleeding vessel, what circumstances indicate that pressure at the site of injury and compression of the supplying arteries in the neighbourhood will avail, the dangers of meddlesome surgery, the propriety "of not searching by operation for the wounded vessel, unless bleeding when the surgeon sees the case," the course of treatment adapted for exceptional instances, and the complications which arise in the progress of cure, are severally illustrated by the history of hospital patients under the charge of the author. Mr. Butcher had some years previously published *A Clinical Lecture on the Treatment of Wounds of the Palmar Arch and of the Arteries in the Vicinity of the Wrist-joint,* in which, having examined the opinions and contrasted the experience of the most eminent English and French authorities, he advocated the pursuance of special treatment, and illustrated the difficulty and danger the surgeon must be prepared to encounter when such cases present themselves. The value of the suggestions in these early contributions cannot be too highly estimated, particularly when it is remembered that in such cases for the surgeon to hesitate is for the sufferer to be lost. Following out his hospital observations and combining with it extensive experience derivable from private practice. Mr. Butcher proceeds to treat "on some rare injuries of joints, the result of accident and disease." Amongst the many cases quoted, one it is desirable to particularize, which combined "compound dislocation of the right wrist-joint with fracture of the radius and ulna immediately above the radio-carpal articulation; fracture of both bones of the left fore-arm above the wrist-joint, with the articulation extensively laid open in front; comminuted fracture of the left thigh-bone, with laceration of the ligaments above the right ankle-joint; and denudation of the frontal bone by a jagged wound from two to three inches in extent, situated above the left brow." This accumulation of injuries, the result of a suicidal leap from a high window, was successfully treated, without the patient bearing any permanent indication of their presence. Its complication of joint injuries occasions an examination of the teaching of Dupuytren, Velpeau, Voillemier, and Nelaton, with the views of Liston and those English authorities who have acquired special reputation. The details of each day's treatment, the advantages of steady persistence in the use of simple remedies, the importance of an artificial substitute for the protection of the joint pending the healing process, the adaptability of the splint modified on Liston's plan, which Mr. Butcher has the merit of introducing to the profession, are each demonstrated, accompanied by clinical observations embracing the known experience on injuries of joints, further exemplified by many cases which, though offering a less complicated subject for treatment, are equally important in the consideration of so difficult a class of accidents. "The excision of the elbow and wrist-joints and the preservative surgery of the hand" forms the subject of an essay the value of which cannot be too highly estimated. Mr. Butcher well observes that "the great practical question which now agitates and convulses the professional mind lies in reference to the operation of excision of diseased bones and joints, as a substitute for amputation." The all-important considerations which operations of this character involve are discussed with a perspicuity which leaves nothing to be desired. The extent of the morbid conditions, as determining the ultimate advantage of preserving the limb, the prognosis of what may be a useful limb, the conservation of its muscular integrity for purposes other than those of the abolished joint, the best method of performing the operation, the dangers that attend and the complications that embarrass it, are each illustrated in cases where, subsequent to excision, the observations of Moreau were confirmed, and a perfectly useful limb, free from deformity, achieved. Mr. Butcher next affords many illustrations of "excision of the wrist-joint and carpus," followed by the happiest results. In treating of this subject, the experience of Fergusson, Simon, and Stanley is examined, for the determination of the conditions which render excision preferable to amputation, when Mr. Butcher renders fitting acknowledgment to those great authorities who have, in the vindication of surgical conservation, best promoted surgical reform. A large number of illustrations are afforded of cases successfully treated by Mr. Butcher, in which limbs whose joints were far advanced in hopeless, though not helpless, disease, have, by the dexterous use of the knife, and the skilful employment of after-treatment, been restored to comparative soundness; the eventuation of many apparently hopeless recoveries justifying the impressive and simple words of Moreau when he observed in reference to results of operations on joints—" If these things seem incredible, they may be easily brought to the test of experiment. I am firmly of opinion that in similar circumstances the issue will be the same."

Passing from these considerations, the important subject of " encephaloid cancer and the cancerous degeneration of warty excrescences" is treated of. The relationship between cancer and fungus hæmatodes is also examined. Many cases are set forth, showing the fearfully rapid and terribly fatal progress of these diseases. Mr. Butcher advocates the excision of such growths as soon as their nature is distinctly ascertained, and observes, "early operative interference, where operation is applicable at all, becomes, if possible, more imperative in that class of malignant diseases" which he describes as "cancerous degeneration of warty excrescences." Mr. Butcher's "critical remarks on the treatment of fractures of the thigh-bone" are familiarly known to European surgeons. His determined advocacy of the employment of his modification of Liston's splint acquires an almost personal interest, from the warmth of his antagonism to Professor Syme, whose practical surgery bears a closer analysis than the logic "north of the Tweed," for, most assuredly Mr. Butcher is unanswerable in his arguments on Professor Syme's objections, and the application of the long splint. Mr. Butcher, in sustaining his logic of argument, also appeals to the logic of facts, and is corroborated by the experience of the greatest living authorities; and further, supported by the successful issue of many examples which show that Professor Syme in seeking to establish as a fixed principle that "extension" in the treatment of fractured thigh was not consistent with "coaptation and immobility," either had been unfortunate in the selection of cases, or has had an experience strangely at variance with that witnessed in the Dublin hospitals, and familiarly known in our metropolis. Mr. Butcher's observations on the treatment of fractures are deserving of the careful perusal of all, especially of those whose position may entail on them the responsibility of determining by successful treatment, whether an accident, admitting of complete cure, is or is not to eventuate in a permanent disability for occupation demanding the use of limbs in their integrity. Many interesting details of operations are afforded, embracing "excision of the knee-joint" and "excision of a large portion of the femur." These, with Mr. Butcher's observations on "fatal bleeding in psoas abscess from ulceration of the cava," "examples illustrative of the application of ligature on the popliteal artery in cases of disease of knee-joint," with further remarks on "the treatment of the suppurative crisis of joints by free incisions," worthily sustain their author's reputation. The next "series" includes many examples of excision of the "carpus," of the knee-joint, of the "elbow," and of the "lower jaw," together with illustrations of Syme's and Pirogoff's operations at the ankle-joint, with a careful analysis of the circumstances which should guide in the adoption of either, and an examination of the relative merits of each. In these criticisms, the experience of Fergusson and other English operators is fairly commented on—Mr. Butcher according to each that share in the great whole to which he is fairly entitled. Mr. Butcher's observations on excision of the upper jaw, and on "the operative measures necessary in complicated hare-lip," are of the highest practical importance. While the cases illustrating the former afford examples of that extraordinary success which by expert hands may be achieved for the removal of those terrible afflictions disease entails, to the latter it is desirable to direct especial attention. Complicated congenital deformities most severely tax the skill of the surgeon. To him is committed the future of the infant, when he is called on by the exercise of his art to supply the deficiencies or remove the deformities of those who,

> "Cheated of feature by dissembling Nature,
> Deform'd, unfinish'd, sent before their time
> Into this breathing world, scarce half made up"—

would, without such interference, be most miserable. In no class of malformation is surgery capable of achieving greater triumphs than in those defective conditions of the palate and lip to which Mr. Butcher directs particular notice. The details of these complications, and the different varieties of malformations, with the treatment which Mr. Butcher suggests as most applicable for each, cannot, on the present occasion, be particularized. The papers are referred to as deserving the careful study of every practitioner on whom the responsibility of such operations is likely to devolve. It may be observed that an early age is advocated as being the most suitable, and promising the best results; and the administration of chloroform is most unhesitatingly condemned, as affording no adequate counterpoise to the great dangers which its use contributes to in all operations about the mouth, especially when the patients are of tender years. The particular instruments Mr. Butcher uses he affords diagrams of. The extraordinary success with which their use has been followed in his hands renders unnecessary any observations in their commendation.

Passing from those operations on the upper jaw, lips, and palate which congenital deficiency renders requisite, Mr. Butcher proceeds to the consideration of many serious affections resulting from disease in adult life and affecting the same structures. In their contrast the surgeon is presented with a strange antagonism, as it were, in the economy of Nature, and a beautiful illustration of her power to adapt her requirements to the exigency of her position. In infant life the object of the operator is to accommodate the defective materials, and to enable art to supply that which deformity has rendered deficient. In adult life the surgeon is called on to remove large masses of disease, involving the framework of the surrounding structures. Nature speedily becomes resigned to the loss, and rewards triumphant surgery by her complete reciprocity in the efforts of successful art. Of course, in reading the details of operations requiring boldness, dexterity, and knowledge, the surgeon must be prepared to include that "genius for cure" which seems to appertain to many. It is undoubtedly true that the labours of one do not bear comparison in results with those of another, the reason resting in causes in which the knife has no part, for when IT is laid down, the real work of the surgeon in such instances only begins. The after-treatment, the power of anticipation, the combating the present, the providing for the future, are the real tests by which the operator must be judged. This great Irish surgeon invites to such an ordeal, when, detailing those principles which must guide, those dangers that may arise, what to do, and what to avoid, he illustrates by works, not words, that which success has corroborated. Mr. Butcher passes to the consideration of the operation of "extirpation of the eye-ball," as successfully carried out in an hospital patient. He discusses the authorities on the subject. The case was one of true scirrhus of the eye-ball, occurring as a primary affection, a disease so rarely met with that its existence was doubted by Mr. Wardropp. Mr. Tyrrell is silent on the subject. Mr. Lawrence has detailed but a single case. In this example no doubt of the nature of the affection existed. Mr. Butcher offers valuable directions in cases in which an artificial eye cannot be made applicable. He writes: "Be the sufferer rich or poor, it is of the utmost importance that extraneous matter floating about in the atmosphere, and the sharp winds of a changeable climate should be guarded off from the delicate and sensitive parts within," and suggests "that after separation of the lids by section at the outer canthus, and after the several steps of the operation to the removal of the organ, not bringing the divided surfaces in contact by adhesive straps or stitches, but permitting the upper lid to slightly overlap the lower, by a little more than the depth of its dorsal cartilage." He further proceeds to suggest remedies proper in case the drooping of the lid should be too great, or it should be inclined to fall inward, either of which occurrences are productive not only of annoyances by extraneous matter, but also of considerable increase of that deformity, which under such circumstances the operation necessarily entails. A very remarkable case is next recorded of "the removal of the entire radius from one articular surface to the other, with nearly all the functions and powers of the limb preserved." Mr. Butcher's own words best express the teaching of the operation:—

"The whole case, in its management from beginning to end, teaches a valuable lesson: how youth will repair accidents of the gravest nature by the vitality of its tissues; how that, even with the severest complications, results may be expected that never could be anticipated in later years; how that when, through additional injury from accidental circumstances, all nature's efforts at repair may be broken up and destroyed, and absolutely a large portion of the solid part of the limb perish by death; yet how that, in youth, this deadened bone, fatal to life if permitted to remain, may, by a bold and dexterous operation, be removed; and how it may be replaced by a substitute; and how the limb may be restored to nearly the full performance of all its functions, *by conservative surgery.*"

In further confirmation of these remarks an example is given of "death of the shaft of the femur from fracture; protrusion of one end of bone; extraction of six inches and a half of it; limb restored to nearly its full length by the application of 'Butcher's splint.'" This case is most interesting, not only as an illustration of the recuperative power of nature in youth—the patient being but thirteen years of age—but also of the advantage of well-adjusted mechanical appliances to assist Nature in her efforts, and to prevent undue trial of those structures she supplies when called on for the purposes of cure. The operations of amputation at the knee-joint, at the knee, and excision of the knee-joint, are next entered on. In detailing the former, Mr. Butcher writes:—

"The flaps being well up before and behind, and the limb carefully steadied, I laid the fine blade of my own saw on the healthy osseous tissue close to the cartilage, but not infringing upon it, and then cut the bone in a curved manner from before backwards, thus securing even a longer stump, more of the bone free of cartilage, and exempt from sharp and irritating edges, better in every way for adaptation to the soft parts. I was the first, I believe, to lay stress upon this method of sawing the bones in certain amputations."

The saw known as " Butcher's saw" must be regarded as one of the most useful instruments in the hands of the surgeon. The profession recognises its value. It is indispensable for the purposes Mr. Butcher specifies. The irritation which not unfrequently retards the healing of stumps after amputation is by its use almost invariably avoided, and the more speedy formation of an available surface for mechanical purposes secured. This instrument is one of many devised by Mr. Butcher, and introduced to the profession with the recommendation of extraordinary success in accomplishing the purposes for which it was designed.

These cases are specially worthy study. They abound with practical suggestions of the first value, more especially to the public officer who may, at a moment's notice, be called on to act in the face of complicated difficulties. In no particular do they fail to impress the reader with the reality of surgery as a science which cannot be considered apart from its exercise as an art, requiring great mechanical skill and adroit manipulation—the former determining what treatment can accomplish most; the latter how that treatment is to have practical effect.

Mr. Butcher proceeds to investigate a class of injuries that unhappily have of late years acquired extraordinary importance, namely, " Deformities after Burns."—Examples are given in which extensive cicatricial deformity, in one instance binding the chin to the sternum, and in the other binding the head to the shoulder, were completely rectified with scarcely a trace of deformity. Perhaps in no class of cases is more difficulty experienced than in the treatment of injuries by fire: at every step a new danger arises. The shock escaped, its immediate secondary effects are scarcely less formidable. The peril of reaction past, the patient enters on a course, the comfort or misery of which too often depends on the capability of the surgeon. The diversified deformities resulting from burns have led to the suggestion of varied operations modified to suit special cases. The plan adopted by Mr. Butcher was novel and successful. It consisted of involving in a large flap the cicatricial tissue and its subcutaneous covering, and free division, by repeated incisions from before backwards, and above downwards, throughout its entire extent. The perfect success which attended the operation is the best argument in favour of its adoption.

The secondary complication of amputations are examined, when the treatment of pyæmia by the abundant exhibition of stimulants, and the free administration of mercury and opium, has the advocacy of Mr. Butcher as that which his large practical experience suggests. The various opinions of pathologists as to the nature of the affection, and the theories in support of their views, ultimately merge into the great question—what is to be done when the disease is present? From whatever cause proceeding, or how produced, Mr. Butcher believes it is best met by the administration and employment of those means and medicines which sustain, while specifically affecting the system. The treatment by ligature of the femoral artery, of elephantiasis Arabum affecting the lower extremity, is illustrated. As a complete restoration to the normal condition was accomplished, we extract the measurement of the diseased as contrasted with the sound, limb. Thigh above knee of sound limb, 15½ inches; of diseased limb, 17½ inches; calf of leg of sound limb, 14 inches; of diseased limb, 19 inches; above the ankle, sound limb, 8 inches; of diseased limb, 16½ inches; above the arch and dorsum of foot, sound limb, 10 inches; of diseased limb, 15½ inches. The details of this case are most interesting. The complete subsidence of the swelling and pain, and the gradual restoration of the powers of the limb, are each day traced, until at length the patient left the hospital able to pursue her occupation as a laundress, without experiencing other inconvenience than such as may result from the precautionary application and use of a bandage ordered never to be dispensed with.

In detailing the steps of the operation Mr. Butcher takes occasion to offer many practical directions respecting the best method of taking up the artery, and examines the complications and difficulties which are so frequently experienced in the attempts to do so. In this, as in the other cases which the Reports contain, the same practical teaching prevails. Unlike a set work, which discusses the approved methods to be adopted in an admitted condition, Mr. Butcher's Reports appeal to the practical knowledge of his readers. They bring him to the bedside of the patient; they point out complications on which routine works are silent; they then teach him what to do; and, finally, instruct him how to do it.

It is not possible within the limits of a review to enter minutely into an examination of the various complications of injury, and the suggestions for their treatment, with which these Reports abound. It is enough to observe they exhaust the subject of operative surgery, and embody the experience of the greatest of Irish authorities. It is but justice to the Irish school that special allusion be made to Mr. Butcher's memoirs *On Excision of the Knee-joint,* which is appropriately dedicated to William Fergusson, Professor of Surgery in King's College, "as a tribute of admiration for his pre-eminent professional ability, especially in the department of conservative surgery, of which he has been the chief promoter in this empire." These essays are so familiarly known as embracing the experience and literature on the subject, that observations respecting them may be limited to the conclusions at which Mr. Butcher arrives from the result of the several cases he has successfully treated. Having detailed the steps of the operation, Mr. Butcher proceeds :—

"Parts being thus divided, the lower flap was detached somewhat downwards, whilst the edge of the knife was kept very closely up to the posterior surface of the tibia and back of the condyles, the immediate connexion of parts being thoroughly freed from their adherence to the line beyond the articular surface in either bone; and this is all that is requisite, as then the finger will detach, by vigorous pressure, the soft parts from the bones to the required extent in this their posterior aspect. By this simple manipulation the artery is not only secured from injury, but it remains undisturbed in its bed, and the popliteal space is not encroached upon, or its anterior wall broken up"— a point Mr. Butcher considers of the first importance. The conclusions at which Mr. Butcher arrives respecting the other steps of the operation are:—"The H incision should be preferred: the patella should be taken away in all cases, whether diseased or not; all bleeding vessels should be tied, or any that have sprung and retracted should be drawn out and secured; while the patient is yet on the operating-table, the limb should be placed in the horizontal position, either by gentle and steady traction, combined with pressure of the cut surfaces of the bones backwards, or, if necessary, by the division of the hamstring tendons. During the adjustment of the bones, great caution should be exercised that their surfaces be throughout their extent in contact, and that no soft parts intervene; the limb should not be disturbed for several days. In cases where large abscesses form in the vicinity of the diseased joint, or up along the thigh, Chassaignac's drainage-tubes may be used with the best hopes of success. The free administration of stimulants and sedatives is imperatively demanded in all cases of excision, regulated to a certain extent by age, sex, temperament, and habit."

In order to accomplish the several ends designed by the operation, Mr. Butcher lays great stress upon the settlement of the limb in a box splint (Butcher's box). This and the careful adjustment of the anterior splint he considers indispensable, to prevent the femur starting forward from the pressure of the body downward.

The excision of the bones, Mr. Butcher in all cases performs with the saw bearing his name. Its special capabilities for such purposes the profession is familiar with.

The value of these Reports is materially increased by the admirable manner in which they have been illustrated. Engravings and lithographs convey, in many instances, fac-similes of the morbid growths and their special characteristics of formation and colour. Frequent allusion is also made to "casts" in the author's possession. It is known that for many years Mr. Butcher has been forming a museum illustrative of surgical diseases. This, which embraces a large number of invaluable and unsurpassable models, is accessible only to a few. We commend to Mr. Butcher the example of the late distinguished Professor Montgomery, who, during his life, placed in the school of his university his several preparations, and so added an additional inducement to foreigners to visit Dublin for the purposes of study.

In thus briefly noticing the contributions of one who has become identified with the Dublin School of Surgery, but a very imperfect *resumé* of his claims to professional distinction has been accomplished. These claims have, in the sister metropolis, been adequately appreciated. The College of Surgeons have called Mr. Butcher to the important position of Chairman of its Surgical Court; and the Dublin University have conferred upon him the highest honour open to them to bestow—their honorary M.D. Our Irish medical brethren occupy a deservedly high position in the estimation of the public and of the profession. In medicine, the works of Graves and Stokes are class-books. Their teaching reveals the degree of excellence to which it is possible for hospital instruction to attain. The several contributions of Corrigan, Hudson, Mayne, Banks, and others rank with the standard treatises of our profession, and proclaim the practical spirit which is characteristic of their medical research. In surgery, who is not familiar with Crampton, Cusack, Porter, Rynd, and others, who have achieved reputations co-extensive with the treatment of suffering throughout the world? Death has been busy in their ranks within the last few years. Great and good men have been called from their labours. Those eminent surgeons particularized no longer live. Their reputations, and the inspiration of their example, are not wanting to their survivors. Mr. Butcher has given expression to both, in placing on record his experience, and affording to the operative surgeon a series of "Reports in Surgery," which have achieved for him a more than European fame.

